# Umbilical Cord-Derived Mesenchymal Stem Cells for the Treatment of Infertility Due to Premature Ovarian Failure

**DOI:** 10.7759/cureus.30529

**Published:** 2022-10-20

**Authors:** Kritika Garg, Sarju Zilate

**Affiliations:** 1 Department of Pharmacology, Jawaharlal Nehru Medical College, Datta Meghe Institute of Medical Sciences, Wardha, IND

**Keywords:** premature ovarian failure (pof), umbilical cord-derived mesenchymal stem cells (ucmsc), infertility, autoimmune disorders, hormone replacement therapy (hrt), stem cell transplantation

## Abstract

Females belonging to the reproductive age group may face challenges regarding infertility or miscarriage due to conditions such as premature ovarian failure (POF). It is the condition that happens when a female's ovaries stop working before she is 40. The majority of the causes of POF cases are idiopathic. Other reasons include genetic disorders (Turner's syndrome, bone morphogenetic protein 15 (*BMP15*) mutation, galactosemia, mutation of forkhead box protein L2 (*FOXL2*), growth differentiation factor-9 (*GDF9*), mutation of luteinizing hormone (LH) and follicle-stimulating hormone receptors (*FSHR*), etc.), enzymatic mutation such as aromatase, autoimmune disorders (Addison's disease, vitiligo, systemic lupus erythematosus, myasthenia gravis, autoimmune thyroiditis, autoimmune polyglandular syndrome, etc.), vaccination, and environmental factors (cigarette smoking, toxins, and infections). Many attempts have been made to treat POF by various methods. Some of the methods of treatment include hormone replacement therapy (HRT), melatonin therapy, dehydroepiandrosterone (DHEA) therapy, and stem cell therapy. Stem cell therapy has proven to be the most efficient form for treating POF as compared to all other options. Umbilical cord-derived mesenchymal stem cells (UC-MSCs) are the best among the other sources of mesenchymal stem cells (MSCs) for the treatment of POF as they have a painless extraction procedure. They have a tremendous capacity for self-repair and regeneration, which helps them in restoring degenerated ovaries. This review includes information on the causes of POF, its efficacious therapeutic approaches, and the impact of transplantation of human umbilical cord mesenchymal stem cells (hUCMSCs) as an option for the therapy of POF. Numerous studies conducted on stem cell therapy prove that it is an effective approach for the treatment of sterility.

## Introduction and background

Premature ovarian failure (POF) affects roughly 1%-3% of females under the age of 40 years. Decreased levels of estrogen, amenorrhea, elevated gonadotropin levels, and the absence of mature follicles are the main characteristics [1]. Some of the causes of premature ovarian failure include autoimmune disorders, genetic diseases, and environmental factors [2]. In most instances, the cause of POF is idiopathic [3]. A decline in the function of the ovaries at an early age can cause a plethora of severe implications on a female's health and can cause psychological consequences [4]. POF has a very diverse clinical presentation and can cause serious health implications, including infertility, osteoporosis, and heart diseases.

Many techniques have been employed to treat POF by using exogenous gonadotropins to induce ovulation [5]. The results of some cases were encouraging, but most of the patients treated with such techniques did not yield a positive impact. The treatment of POF in females should be started early to prevent severe consequences later in life [6]. The only reliable approach available in females for the treatment of POF was ovum donation to establish a pregnancy, but this method was not very well received by people with the disadvantage of maternal gene abstinence in the developing embryo. Furthermore, this approach was not very acceptable for many couples due to religious and cultural differences. Many attempts at ovarian stimulation have been in vain, which is a significant reason for severe psychological consequences in patients [7].

Mesenchymal stem cells (MSCs) have presented immense capability for managing various diseases by developing new treatment techniques [8]. It is a unique and upcoming cell-based therapy and is increasingly gaining ground in clinical practice as stem cells can regenerate and have the potential to self-repair; they are now being used to treat ovarian failure and infertility. They have many prominent advantages other than ample supply, painless extraction, and a fast self-regeneration of human umbilical cord mesenchymal stem cells (hUCMSCs), such as the potential to divide into the three layers of germ cells that help in the function and growth of other cells. Several methods have been developed for isolating these cells from Wharton's jelly, arteries, or veins. These mesenchymal stem cells can quickly be transferred to the affected pathological areas through conventional methods [9].

## Review

Etiology

The primary follicle developing into secondary follicles and then further into the antral follicle is a normal process known as folliculogenesis. This process is then followed by ovulation [10]. If there is any disruption in this natural process, it will lead to premature ovarian failure [11]. Most cases accounting for nearly 75% of POF are idiopathic [12]. Table 1 includes a list of other causes [2].

**Table 1:** Premature ovarian failure etiology POF: premature ovarian failure; *FOXL2*: forkhead box protein L2; *FOXO3A*: forkhead box O3; *FSHR*: follicle-stimulating hormone receptor

Causes of POF	Examples
Genetic	Turner syndrome, mutation of *FOXL2* and *FOXO3A*, and *FSHR* mutation
Autoimmunity and ovarian function	Autoimmune thyroiditis, Addison's disease, diabetes mellitus, myasthenia gravis, and autoimmune polyglandular syndrome
Radiotherapy and chemotherapy	Cyclophosphamide and nitrogen mustard
Infection	Mumps orchitis
Enzymatic	Aromatase deficiency and 17α-hydroxylase deficiency
Environmental toxins	Cigarette smoking and ionizing radiation

Genetic Diseases

Genetic changes have been seen in females suffering from POF at a single autosomal locus and X-linked loci. Many genetic diseases such as Turner's syndrome, X-linked disorders, and gene mutation in autosomal chromosomes were detected in the cases of POF [13]. Turner's syndrome presents as the main X chromosomal abnormality causing primary amenorrhea due to ovarian degeneration. Some other mutated genes involved in POF development are follicle-stimulating hormone receptor (*FSHR*), forkhead box protein L2 (*FOXL2*), forkhead box O3 (*FOXO3A*), growth differentiation factor-9 (*GDF9*), etc. [14]. Fragile X syndrome is the trisomy of the X chromosome resulting in primary ovarian insufficiency. Turner's syndrome is characterized cytogenetically by the monosomy of the X chromosome (45,X) [15]. The clinical presentation is gonadal degeneration with primary amenorrhea, infertility, cubitus valgus, pigmented nevi, webbed neck, peripheral lymphedema at birth, coarctation of the aorta, and short stature in these phenotypes of females [16]. Evidence suggests that short stature and failure of ovaries in females might be mapped to another part of the X chromosome, which proves their direct relation [17]. *FOXL2* is a domain transcription factor of a forkhead shape, and any mutation seen in this can cause blepharophimosis-ptosis-epicanthus inversus syndrome (BPES) type I, which can cause POF in females [15]. The mutation of *FOXL2* in mice ovaries, follicular degeneration, and subsequent atresia are caused due to the failure of differentiation of granulosa cells [18]. *FOXL2* has a regulatory role in folliculogenesis, and any mutation in this mechanism can disrupt the normal development of the ovarian follicle [15]. During the reproductive period of a female, the production of progesterone by the theca cells is regulated by follicle-stimulating hormone (FSH) and luteinizing hormone (LH) [19]. The *FSHR* is of significant importance in patients suffering from POF. The inadequacy of this receptor can decrease its FSH-binding ability or the activation of the signal transduction pathways involved [20].

Autoimmunity and Ovarian Function

Abnormality in recognition by the immune system, known as autoimmunity, can also lead to POF [2]. Evidence shows that nearly 30%-50% of the POF cases were associated with autoimmune disorders. The most common disorder was thyroid-related disorders such as Hashimoto's thyroiditis, hypothyroidism, and hyperthyroidism [21]. The second most frequent condition related to the cases of POF is adrenal autoimmune disorders [22]. Its smaller percentage is also associated with other disorders such as systemic lupus erythematosus, myasthenia gravis, vitiligo, celiac disease, rheumatoid arthritis, and autoimmune polyglandular syndrome [23].

Radiotherapy and Chemotherapy

Chemotherapy and radiotherapy are of great importance for treating specific malignancies. It is one of the leading reasons of POF. Chemotherapy treatment for breast tumors, myeloid leukemia, non-Hodgkin's lymphoma and Hodgkin's lymphoma can cause degeneration and failure of the ovaries in nearly 50% of the cases [24]. Exposure of the ovaries to alkylating agents such as cyclophosphamide and procarbazine for chemotherapy can lead to POF [25]. Interestingly, no discernible rise in birth abnormalities has been reported in subsequent pregnancies for females receiving radiation therapy or chemotherapy, provided their fertility is retained [26].

Infection

Earlier reports showed the occurrence of a viral infection such as varicella in the past medical history of the patients of POF [27]. Researchers found that, during an epidemic, the patients who suffered from mumps had an increased risk of oophoritis of 3%-7%, with an intermittent onset of infection [28]. Immunocompromised patients with diseases such as AIDS and lymphoma or patients receiving a transplant have been noted to have cytomegalovirus-related oophoritis [28]. Although POF has been linked to infections with shigella, varicella, malaria, and tuberculosis, a cause-and-effect connection has not been proved. Only clinically critical situations would warrant the use of screening for these illnesses. The actual frequency of ovarian failure related to a viral disease is unknown [29].

Enzymatic

Any defect in the enzymes such as 17α-hydroxylase, cholesterol desmolase, and aromatase can cause histological and clinical irregularities such as the disruption of estrogen synthesis, causing a delay in the onset of puberty, primary amenorrhea, and raised levels of gonadotropin [30]. 17α-Hydroxylase deficiency in patients can cause hypertension and failure in the formation of adrenal and ovarian steroids, leading to ovarian failure [29]. On physical examination, the ovaries of these patients may be palpable, and they could be at a risk of ovarian torsion and infarction.

Environmental Toxins

Environmental toxins such as smoking, insecticides, plastic material, and radiation can lead to POF and, thus, infertility [2]. According to widely conducted epidemiological studies, smoking can decrease the menopausal age by 1-2 years [31]. Additionally, cigarettes have a dose-dependent impact on fertility that starts at one pack per day. Smoking can disrupt the function of the ovaries in multiple ways. There is a decline in follicle aging, and the germ cells are affected by the harmful polycyclic hydrocarbons in cigarette smoke [32]. Nicotine inhibits the aromatase enzyme, which causes a decline in estradiol production [33].

Therapeutic strategies

The initial step for treating premature ovarian insufficiency is to relieve the signs of deficiency of estrogen and reduction in the risk of cardiovascular diseases and maintain bone density by replacement therapy of estrogen and progesterone. A proper past medical history of the patient must be appropriately devised for hypothyroidism, adrenal defects, and autoimmune disorders. Family history should include information about POF, any genetic defects, and ovarian insufficiency at all stages. There should be a confirmation by the repeat FSH test in laboratory examination. Unfortunately, an average FSH level does not indicate an enhancement in the ovarian function because fertility is still impaired in these females [34]. Many attempts have been made to treat POF, but all these methods were not entirely successful. Transplantation of stem cell was the most effective treatment of all the ways [2]. Various therapeutic strategies have been listed in Table 2 [2].

**Table 2:** Therapeutic strategies

Treatment strategies	
Hormone replacement therapy (HRT)	Serum estradiol, estrogen replacement therapy, progesterone replacement therapy, androgen replacement therapy, transdermal estrogen replacement, piperazine estrone sulfate, Duphaston, norethindrone, micronized progesterone, and medroxyprogesterone
Melatonin therapy	
Dehydroepiandrosterone therapy	
Immunomodulation	Monoclonal antibodies, immunosuppressors like corticosteroid, and extra-embryonic stem cell
Stem cell therapy	Mesenchymal stem cell, ovarian stem cell, and induced pluripotent stem cell

Hormone Replacement Therapy (HRT)

There is a need for long-term hormone replacement to relax menopausal symptoms (such as the instability of vasomotor functions, mood, sexual dysfunction, and skin problems) and to stop long-term issues due to deficiency of estrogen, which can lead to osteoporosis [35]. The maintenance of LH levels, which sustain fertility by hormonal therapy, is known to cause a reduction in the cases of POF [36]. There is improvement and restoration of endothelial function after six months of hormonal therapy [37]. Multiple researches have demonstrated that apart from its overall positive impacts, hormone replacement therapy (HRT) boosts growth hormone (GH) formation in postmenopausal females [38].

Melatonin

Studies show evidence of increased levels of gonadotropin, thyroid function, and fertility and menstruation recovery by melatonin therapy [2]. Recent studies show several tissues, including reproductive tissues such as the ovary and placenta, produce melatonin [39]. It has been seen that ovulation can be supported by melatonin. Because melatonin controls the immune system, it is being utilized to treat cancer, which stimulates humoral immunity and cell-mediated immunity [6].

Dehydroepiandrosterone (DHEA)

In a female suffering from POF, DHEA is frequently administered as there is a low ovarian reserve and decreased response to ovary stimulation. DHEA is an endogenous steroid formed in females from the zona reticularis and the ovarian theca cells [40]. It is a crucial prohormone for ovarian follicular steroidogenesis [41]. Earlier reports have shown that DHEA administration in POF cases can increase pregnancy rate and reduce the rate of miscarriages, making intravenous fluid (IVF) treatment more efficacious [6].

Immunomodulation

Immunomodulation proves to be an effective method of the treatment of POF in cases of ovarian damage by autoimmune diseases [42]. In POF, the autoantibodies seen are steroid-producing cell antibodies; these antibodies help to bind to corpus luteum, cells of granulosa, and theca cells [2]. Based on that, monoclonal antibodies and corticosteroids are used as a treatment for immunosuppression [43,44].

Stem Cell Therapy

Under right circumstances, stem cells are distinct pluripotent cells with the capacity to divide and transform into any cellular tissue. Stem cells have the property of self-regeneration and self-repair, which is why they are appropriate for this treatment. They can differentiate into the three germ layers leading to degenerated ovaries' restoration [45]. Recent studies on animal models have proven stem cell therapy to be an efficient treatment for premature ovarian failure as it can restore the structure and function of the ovary among the other methods [46]. For the therapy of POF, various stem cell types include the following.

Extra-embryonic Stem Cells

Stem cells from amniotic ﬂuid are multipotent and are derived from the extra-embryonic layer. Their proliferation occurs more quickly from mesenchymal stem cells [13]. Transplantation of stem cells from the ﬂuid of the amniotic cavity inhibits the degeneration of the follicles and maintains their function [2].

Mesenchymal Stem Cells

Many researchers have proved that transplantation of mesenchymal stem cells (MSCs) is the most eﬃcient cellular method for POF treatment [47]. These stem cells can stimulate many mechanisms such as trophic, paracrine, and differentiation. This helps to restore the tissue damage [48]. Bone marrow mesenchymal stem cells (BM-MSCs) have great potential to proliferate and differentiate [49]. Therefore, they are known as the "gold standard" of historically accepted MSCs. Recent research has shown that mesenchymal stem cells can also be derived from various sources in the body, such as the umbilical cord, bone marrow, amniotic ﬂuid, placenta, skin, and adipose tissue [48]. Umbilical cord-derived mesenchymal stem cells (UC-MSCs) have an exceptional combination of prenatal and postnatal stem cell traits [50]. This study primarily focuses on this subject.

Human umbilical cord mesenchymal stem cell (hUCMSC) therapy

hUCMSC therapy is an emerging method for treating premature ovarian failure. Unlike the other sources of mesenchymal stem cells, it has a relatively easier, painless extraction method; multipotency; and infinite self-repair properties [50]. Umbilical cord-derived mesenchymal stem cells can migrate into areas of injured tissue or inflammation and develop into three separate germ layers, helping in tissue healing [49]. These cells show three significant effects: tissue repair, immune response modulation, and anticancer abilities [2]. By analyzing flow cytometry, it was found that umbilical cord-derived mesenchymal stem cells express matrix receptors (CD44 and CD105) and integrin markers (CD29 and CD51) but not hematopoietic markers (CD34 and CD45) [51].

The Importance of hUCMSC

The immense therapeutic potential of human mesenchymal stem cells (MSCs) has recently made it a promising method of treatment [2]. Due to no immune response against them, painless collection procedure of the abandoned umbilical cord and less ethical issues are the significant advantages of hUCMSC compared to various supplementary sources [52]. When cultivated alongside injured human endometrial stromal cells (ESCs), hUCMSC considerably lowers the apoptosis rate and increases ESC proliferation [53]. In one of the studies on a rat model, it was found that UC-MSC transplantation increased follicular growth, and also, the levels of progesterone and estradiol were greatly enhanced [2]. The number of functioning follicles and the regular synthesis of hormones are both increased by transplantation. These findings provide theoretical foundations for using stem cells to treat POF and further support the rise in the regeneration of the tissue and growth factors of the cell [54]. It was also observed that mesenchymal stem cells migrate to the damaged tissues and restore them by modifying the immune system and secreting growth factors. Instead of direct development into germ cells, cytokines produced by hUCMSCs via the paracrine route are substantially responsible for improved ovarian function in the rat model of premature ovarian failure (POF) [55].

Stem cell can be derived from various sources such as the embryo, fetus, and bone marrow. Embryonic stem cells (ESCs) are a great candidate for the stem cell therapy due to their fast renewal rate, but their clinical use is highly limited as they may lead to teratomas. The adult stem cells such as bone marrow stem cells have been successfully used for autologous purposes, but they were sometimes limited because of changes related to age and numbers. In comparison to these, UC-MSCs have proven to be better as they have a gene profile similar to that of ESCs and have a faster rate of regeneration than the BM-MSCs [56]. The umbilical cord, which was once considered to be a medical waste, is now recognized as a promising source of MSCs as they have a noninvasive procedure of collection and are furthermore not burdened by ethical problems [57]. Because of their immunomodulatory properties, UC-MSCs have also garnered a lot of attention. According to a number of studies, MSCs produced from the umbilical cord are more primitive, proliferative, and immunosuppressive than their adult counterparts. UC-MSCs have an innate ability to differentiate into oocyte-like cells (OLCs), with zona pellucida-like layer, and thus have shown to be an effective treatment for infertility through various researches [58].

## Conclusions

There is a significant effect on the mental and physical well-being of the patients facing the challenges caused by premature ovarian failure. Hormone replacement therapy is typically used by females suffering from POF to lessen the symptoms of estrogen insufficiency, but it is not particularly successful. Thus, there is a need for better treatment options to resolve this problem. Many attempts have been made through various research types to find the most effective method to treat POF. Stem cell therapy was seen as the most efficient method for treating POF compared to the other multiple trials for treatment conducted. Mesenchymal stem cells from the abandoned umbilical cord could be used to treat POF and infertility since they have the capacity for self-repair and regeneration, as well as the ability to enhance the function of ovaries, increase the number of follicles, raise the levels of sex hormone, and decrease the apoptotic rate of granulosa cell. This shows that mesenchymal stem cells derived from the umbilical cord have great potential as compared to some of the sources. Although UC-MSCs are still not very much integrated into the clinical practice, evidences from recent studies suggest that they may prove to be highly effective for the treatment of several diseases. This review article emphasizes the importance of human umbilical cord mesenchymal stem cells in stem cell therapy as the preferred treatment by several researchers.
